# 

**Published:** 1873-12

**Authors:** 


LIST OF CASH PAYMENTS TO THE RECORD.

| | |
|---|---|
| Dr. L. R. Lincecum, Texas | $2 50 |
| Dr. H. R. Hopkins, N. Y. | 2 50 |
| Dr. Albert Fairfax, Va. | 1 00 |
| Dr. J. A. Caldwell, N. C. | 2 50 |
| Dr. G. E. Harris, Ala. | 2 50 |
| Dr. C. G. Stovall, Ala | 2 50 |
| Dr. G. E. Susadorff, Ga. | 2 50 |
| Dr. W. R. McClain, Ga. | 2 50 |
| Dr. J. F. Wootten, Ga. | 2 50 |
| Dr. W. M. Charters, Ga | 2 50 |
| Dr. R. Browne, N. C. | 2 50 |
| Dr. Geo. A. Dyer, Ind. | 2 50 |
| Dr. J. N. Cheney, Ga. | 2 50 |
| Dr. A. S. Campbell, Miss | 2 50 |
| Dr. J. W. Drewry, N. C. | 2 50 |
| Dr. J. G. Brown, Ga. | 2 50 |
| Dr. J. W. Baker, La. | 2 50 |
| Dr. T. W. Keen, N. C. | 2 50 |
| Dr. J. W. Booth, N. C. | 2 50 |
| Dr. W. E. Brock, Ga., bal | 50 |
| Dr. R. A. Shinpock, N. C. | 2 50 |
| Dr. A. Broach, Tenn. | |
| Dr. Thomas H. Edwards, Fla. | 2 50 |
| Dr. B. M. Walker, N. C | 2 50 |
| Dr. W. W. Ward, N. C. | 2 50 |
| Dr. M. B. Pollard, La. | 2 50 |
| Dr. A. J. Sewell, Ga. | $2 50 |
| Dr. F. M. Thomas, La. | 2 50 |
| Dr. J. C. Terrell, Miss. | 2 50 |
| Dr. W. B. Sykes, Ga. | 2 50 |
| Dr. E. Gary, Ga. | 2 50 |
| Dr. G. M. Boynton, Ala. | 2 50 |
| Dr. Daniel Haygood, Ala. | 2 50 |
| Dr. T. C. Collins, Ala. | 2 50 |
| Dr. F. Walker, Ga. | 2 50 |
| Dr. B. H. Bates, S. C. | 2 50 |
| Dr. J. N. Taylor, La. | 2 50 |
| Dr. E. H. W. Hunter, Ga. | 2 50 |
| Dr. J. B. Williams, N. C. | 2 50 |
| Dr. J. R. Jones, Miss. | 2 50 |
| Dr. William White, Va. | 2 00 |
| Dr. W. J. Reese, Ga. | 2 50 |
| Dr. J. Brandon, Texas. | 2 50 |
| Dr. F. R. Van Eaton, Miss. | 2 50 |
| Dr. R. D. Arnold, Ga. | 2 50 |
| Dr. W. S. Posey, Texas. | 2 50 |
| Dr. N. G. Howard, Ga. | 2 50 |
| Dr. W. H. Hall, Ga. | 2 50 |
| Dr. J. W. Bailey | 2 50 |
| Dr. George Field, N. C. | 2 50 |
| Dr. W. W. Tison, Ala | 2 50 |

The above list of names are those who have been received up to the time of issuing the journal. If we have failed to credit any, they will do us the favor to inform us of the fact. Those who send money after this for 1873, acknowledgement will be made in January number, 1874, or by letter.

### The Southern Medical Record.

EDITED BY

T. S. POWELL, M. D.,

AND

W. T. GOLDSMITH, M. D.

### ASSOCIATE EDITORS.

GEORGIA.

Robert Battey, M. D.,

R. C. Word, M. D.,

W. L. Kirkpatrick, M. D.,

James A. Long, M. D.

W. L. M. Harris, M. D.

H. L. Battle, M. D.

W. C. Musgrove, M. D.

L. B. Bouchelle, M. D.

John C. Drake, M. D.

E. F. Knott, M. D.

Thomas Burdell, M. D.

E. H. W. Hunter, M. D.

V. S. Hitt, M. D.

J. E. Pope, M. D.

J. A. Hunnicutt, M. D.

A. H. Brantley, M. D.

J. J. Knott, M. D.

J. C. Wisdom, M. D.

W. W. Leake, M. D.

NORTH CAROLINA.

J. R. Hughes, M. D.

S. S. Satchwell, M. D.

A. B. Pierce, M. D.

H Kelly, M. D.

Geo. A. Foote, M D,

E. A. Anderson, M. D.

H. T. Bahnson, M. D.

Willis Alston, M. D.

Thos. F. Wood, M. D.

N. J. Pittman, M. D

MISSISSIPPI.

C. B. Ga laway, M D.

Edward Lea, M. D.

S. V D. Hill, M. D.

W. B. Harvey, M. D.

J. W. M. Shattuck, M. D.

E. T. Henry, M. D.

T. P. Lockwood, M. D.

Robert Kells, M. D.

VIRGINIA.

Charles S. Lewis, M. D.

John H Claiborne, M. D.

F. Horner, Jr., M. D.

R. J. Preston, M. D.

A. S. Payne, M. D.

J. E. Lockridge, M. D.

J. S. Wharton, M. D.

Wm. O. Owen, M. D.

Sam'l P. Ripley, M. D.

Benj. Blackford, M. D.

E. A. Drewry, M. D.

Rob B. Tunstall, M. D.

W. F. Barr, M. D.

L. B. Anderson, M. D.

S. L. Jepson, M. D., W. Va.

J. M. Lazzell, M. D.

SOUTH CAROLINA.

E. T. McSwain, M. D.

G. M. Rivers, M. D.

TENNESSEE.

J. D. Tucker, M. D.

Swan M. Burnett, M. D.

TEXAS.

S. M. Welch, M. D.

T. J Heard, M. D.

W. A. Heard, M. D.

A. R Kilpatrick, M. D.

ARKANSAS.

A. D. Hodge, M D.

J. K. Hodge, M. D.

ALABAMA.

J. C. Knox, M. D.

Geo. F. Taylor, M. D.

J. B. [illegible]arnett, M. D.

S. M. Hogan, M. D.

D. S. Hopping, M. D.

J. T. Searcy, M. D.

S. F. Hill, M. D.

### CORRESPONDING EDITORS:

Ely Van DeWarker, M. D., New York,

John H. Weir, M. D., Illinois,

Thos. J. Gallaher, M. D., Pennsylvania,

Robert Robson, M. D., Indiana,

C. C. F. Gay, M. D., New York,

Surgeon of Buffalo General Hospital.

W. T. Taylor, M. D., Kentucky,

B. W. Stone, M. D., Kentucky,

Assistant Physician Western Lunatic Asylum.

James Tyson, M. D., Philadelphia, Pa.,

W. W. Leen, M. D., Philadelphia, Pa.,

M. H. Henry, M, D , New York.

Surgeon to N. Y. Dispensary, Dep't of Venerial and Skin Diseases.

Wm. R. Whitehead, M. D., Colorado Territory,

A. Patton, M. D., Indiana,

Benjamin Howard, M. D., New York,

Charles Kearns, M. D., Kentucky,

S. C. Bussey, M. D., Washington, D. C.,

A. W. Hobson, M. D., Arkansas,

A. W. Calhoun, M. D., now Berlin, Prussia.

T. Curtis Smith, M. D., Ohio.

Geo. Strawbridge, M. D., Philadelphia, Pa.

☞ All business Letters should be addressed to POWELL & GOLDSMITH, Post Office Box 527, Atlanta, Ga.

